# *SNX10* gene mutation leading to osteopetrosis with dysfunctional osteoclasts

**DOI:** 10.1038/s41598-017-02533-2

**Published:** 2017-06-07

**Authors:** Eva-Lena Stattin, Petra Henning, Joakim Klar, Emma McDermott, Christina Stecksen-Blicks, Per-Erik Sandström, Therese G. Kellgren, Patrik Rydén, Göran Hallmans, Torsten Lönnerholm, Adam Ameur, Miep H. Helfrich, Fraser P. Coxon, Niklas Dahl, Johan Wikström, Ulf H. Lerner

**Affiliations:** 1 Department of Medical Biosciences, Medical and Clinical Genetics, Umeå University, 901 87 Umeå, Sweden; 2 Department of Immunology, Genetics and Pathology, Science for Life Laboratory, Uppsala University, 751 85 Uppsala, Sweden; 3 Centre for Bone and Arthritis Research, Department of internal medicine and clinical nutrition, Institute of Medicine, Sahlgrenska Academy, University of Gothenburg, 405 30 Gothenburg, Sweden; 4 Arthritis and Musculoskeletal Medicine Programme, Institute of Medical Sciences, University of Aberdeen, Foresterhill, Aberdeen, AB25 2ZD UK; 5 Pediatric Dentistry, Department of Odontology, Faculty of Medicine, Umeå University, 901 87 Umeå, Sweden; 6 Department of Pediatrics, Umeå University, 901 87 Umeå, Sweden; 7 Department of Mathematics and Mathematical Statistics, Computational Life science Cluster (CLiC), Umeå University, 901 87 Umeå, Sweden; 8 Department of Biobank Research, Umeå University, 901 87 Umeå, Sweden; 9 Department of Surgical Sciences, Radiology, Uppsala University, 751 85 Uppsala, Sweden; 10 Molecular Periodontology, Department of Odontology, Faculty of Medicine, Umeå University, 901 87 Umeå, Sweden

## Abstract

Autosomal recessive osteopetrosis (ARO) is a heterogeneous disorder, characterized by defective osteoclastic resorption of bone that results in increased bone density. We have studied nine individuals with an intermediate form of ARO, from the county of Västerbotten in Northern Sweden. All afflicted individuals had an onset in early infancy with optic atrophy, and in four patients anemia was present at diagnosis. Tonsillar herniation, foramen magnum stenosis, and severe osteomyelitis of the jaw were common clinical features. Whole exome sequencing, verified by Sanger sequencing, identified a splice site mutation c.212 + 1 G > T in the *SNX10* gene encoding sorting nexin 10. Sequence analysis of the *SNX10* transcript in patients revealed activation of a cryptic splice site in intron 4 resulting in a frame shift and a premature stop (p.S66Nfs * 15). Haplotype analysis showed that all cases originated from a single mutational event, and the age of the mutation was estimated to be approximately 950 years. Functional analysis of osteoclast progenitors isolated from peripheral blood of patients revealed that stimulation with receptor activator of nuclear factor kappa-B ligand (*RANKL*) resulted in a robust formation of large, multinucleated osteoclasts which generated sealing zones; however these osteoclasts exhibited defective ruffled borders and were unable to resorb bone *in vitro*.

## Introduction

In healthy individuals, normal bone homeostasis is maintained by balancing bone resorption by osteoclasts with new bone formation by osteoblasts. Osteopetrosis constitutes a heterogeneous group of rare disorders characterized by increased bone mass caused either by an inability to form osteoclasts or by loss of osteoclast function^1–4^. Bone resorbing multinucleated osteoclasts are formed by the differentiation and fusion of mononuclear hematopoietic precursor cells in the monocyte lineage. Their differentiation is mediated by macrophage colony-stimulating factor (M-CSF), which is required for proliferation and survival of the mononucleated cells, and receptor activator of nuclear factor kB ligand (RANKL), which triggers differentiation along the osteoclastic lineage. The main clinical features of osteopetrosis are an increased bone density with brittle bones and multiple fractures, narrowing of foramina in the skull with neurological symptoms, and reduced bone marrow cavities leading to impaired hematopoiesis, anemia and recurrent infections^4, 5^.

Autosomal recessive osteopetrosis (ARO) comprises the infantile, or “malignant” form, and a less severe intermediate form (IARO). In malignant ARO, the skeletal defects have an onset before birth and the resulting nerve damage due to entrapment is irreversible. Treatment with hematopoietic stem cell transplantation (HSCT) is critical in order to prevent disease progression and early death^4, 6^. To date, at least 10 genes coding for osteoclast function or osteoclast differentiation have been demonstrated to be causally involved in the pathogenesis of osteopetrosis^1–4^. The incidence of ARO is estimated to be 1 in 250,000 births, but can be much higher in isolated consanguineous populations^7–9^.

Recently, a missense mutation in the gene encoding sorting nexin 10 (*SNX10* [MIM614780]) was shown to be the cause of ARO (OPTB8 [MIM615085]) in consanguineous families of Palestinian origin^10^. Since then, different mutations in the *SNX10* gene have been reported in osteopetrosis patients of diverse ethnicities^11, 12^. At the initiation of the present study, we were aware of several cases with osteopetrosis of unknown origin in the county of Västerbotten, Sweden. A genealogy study revealed that the patients belonged to the same family. We therefore performed exome sequencing with the aim to find the pathogenic sequence variant causing the disease and, in addition, performed cellular and functional studies to assess the role of the mutation in bone remodeling. During the course of our studies, three patients with osteopetrosis from the Västerbotten County were reported to have a mutation in the *SNX10* gene^12^. We confirmed that all osteopetrosis patients in the Västerbotten County carry the same *SNX10* mutation. Here, we describe the clinical features and natural course of the disease in the largest cohort of patients with IARO reported thus far. We identified the disease-causing mutation in *SNX10*, as well as its origin and age and report detailed functional analyses on osteoclasts carrying this pathogenic sequence variant.

## Results

### Subjects

We studied nine patients, five males and four females, with IARO from the county of Västerbotten in Northern Sweden. The clinical data are summarized in Table 1.

**Table 1:** Clinical features of nine individuals with a splice site mutation in the *SNX10* gene, and intermediate autosomal recessive osteopetrosis (IARO).

*Patient number*	1	2	3	4	5	6	7	8	9
*Gender*	Male	Female	Male	Female	Female	Male	Male	Male	Female
*age* (*year*)	47	43	46	35	24	19	15	12	10
Birth length, weight, ^A^OFC	51 cm	50 cm	52 cm	^B^NA	48 cm	52 cm	NA (±0SD)	NA	48 cm
	4000 g	3620 g	3910 g	3110 g	2930 g	3500 g	3094 g	NA	3305 g
	34 cm	NA	NA	NA	NA	34.5 cm	NA	NA	35 cm
Final height (age year) ^C^(SD)	132 cm	149 cm	160 cm	159 cm	NA	NA	Normal growth, after ^D^HSCT	—	—
	(17)(−6.7)	(20)(−2.5)	(20)(−2.9)	(19)(−1.1)					
Age at onset	Early infancy	Early infancy	Early infancy	Early infancy	Early infancy	Early infancy	Early infancy	Early infancy	Early infancy
Age at diagnosis, ^E^symptoms	7 weeks. Optic atrophy. Macrocephaly. Anaemia (^F^Hb 6.64 mmol/l)	5 months. Optic atrophy. Macrocephaly 46 cm. Anaemia (Hb 6.09 mmol/l)	2 years. Optic atrophy. Macrocephaly	6 months. Optic atrophy. Macrocephaly 44.5 cm	19 months. Optic atrophy. Macrocephaly 50 cm	12 months. Optic atrophy, blindness	28 months. Optic atrophy. Macrocephaly 55.5 cm. Anaemia (Hb 6.33 mmol/l)	Days, disease known in the family	36 months. Retained teeth. Nasal congestion. Optic atrophy. Macrocephaly. Anaemia (Hb 6.6 mmol/l)
Stem cell transplantation	—	—	—	—	—	—	HSCT at the age of 33 month	—	—
Deceased (age)	—	—	—	35 years	—	19 years	—	12 years	10 years
Neurological symptoms	Blindness. Impaired hearing, unilateral profound deafness. Facial paralysis	Impaired vision, (blind on one eye). Impaired hearing. Facial paralysis	Impaired vision, (blind on one eye). Impaired hearing, tuba aperta. Facial paralysis	Blindness. Impaired hearing. Facial paralysis	Impaired vision and hearing. Foramen magnum stenosis, op decompression at the age of 14	Blindness. Profound deafness. Intellectual disability	Impaired vision	Blindness. Impaired hearing. Died due to foramen magnum stenosis at the age of 12	Blindness. Hydrocephalus, ^G^VP-shunt. Decompression op of the brainstem at the age of 8, died due to compression of a postsurgical cephalocele
Haematological symptoms	Anaemia, hepato-splenomegaly, splenectomy at the age of 5. Blood transfusions.	Anaemia, hepato-splenomegaly. Blood transfusions.	Anaemia, hepato-splenomegaly. Blood transfusions.	Anaemia, hepato-splenomegaly. Blood transfusions Died due to septicaemia originating from osteomyelitis.	Almost normal haematopoiesis.	Anaemia, hepato-splenomegaly.	Normal haematopoiesis.	Anaemia, hepato-splenomegaly, splenectomy at the age of 9. Blood transfusions.	Anaemia, hepato-splenomegaly, splenectomy at the age of 5. Blood transfusions.
Eruption of primary teeth (month), and teeth development	NA Malformed and retained	At the age of 4-6 months, ten primary teeth Malformed and retained	At the age of 6 months, five primary teeth Malformed and retained	NA Malformed and retained	At the age of 6 months, 12 primary teeth Malformed and retained	NA Malformed and retained	At the age of 28 months, two primary teeth Malformed and retained but a lot better than patients not transplanted	At the age of 13 months, six primary teeth Malformed and retained	At the age of 31 months, eight primary teeth Malformed and retained
^H^ONJ (year)	At the age of 6. Osteonecrosis of the ear canal, and the skull with associated epidural abscess	At the age of 13. Osteonecrosis of the ear canal	At the age of 30. Osteonecrosis of the ear canal	ONJ (age NA)	At the age of 18.	At the age of 12. Died due to septicaemia originating from ONJ	None	None	None
Airway symptoms	Narrow nasal, and pharynx cavities Tracheostomy at the age of 22. ^I^CPAP during night	Early nasal obstruction, before the age of 5 months. CPAP during night	Narrow nasal, and pharynx cavities CPAP during night since the age of 35.	Narrow nasal, and pharynx cavities	Narrow nasal, and pharynx cavities	Narrow nasal, and pharynx cavities	None	Narrow nasal, and pharynx cavities Apnoea during the night Tracheostomy at the age of 9.	Nasal obstruction, before the age of 12 month, adenoidectomy Tracheostomy at the age of 8.
Bone fractures	Multiple fractures, pseudoarthrosis	Multiple fractures, pseudoarthrosis	Multiple fractures, pseudoarthrosis	Multiple fractures, pseudoarthrosis	Multiple fractures	Multiple fractures	None	Multiple fractures	Few fractures
Other features	Exophthalmos keratitis, endophthalmitis, enucleation of one eye. Ear canal stenosis, Kidney tumour, Urinary stones, Weight loss, Wheelchair bound	Exophthalmos Drooling, treated with Botox, Wheelchair bound	Exophthalmos, Bilat pes calcaneovalgus, Hypertension, Walking frame	Exophthalmos Media otitis, Wheelchair bound	Exophthalmos, Mitral valve insufficiency, Wheelchair bound	Exophthalmos Inguinal hernia, Wheelchair bound	Exophthalmos	Exophthalmos, Drooling, Anosmia	Exophthalmos, Severe back pain

^A^OFC = occipital-frontal circumference. ^B^NA = Not available. ^C^SD = Standard deviations. ^D^HSCT = hematopoietic stem cell transplantation. ^E^Symptoms that lead to the diagnosis. ^F^Hb = hemoglobin. ^G^VP = ventriculo-peritoneal shunt. ^H^ONJ = osteonecrosis of the jaw. ^I^CPAP = continuous positive airway pressure.

**Figure 1:**
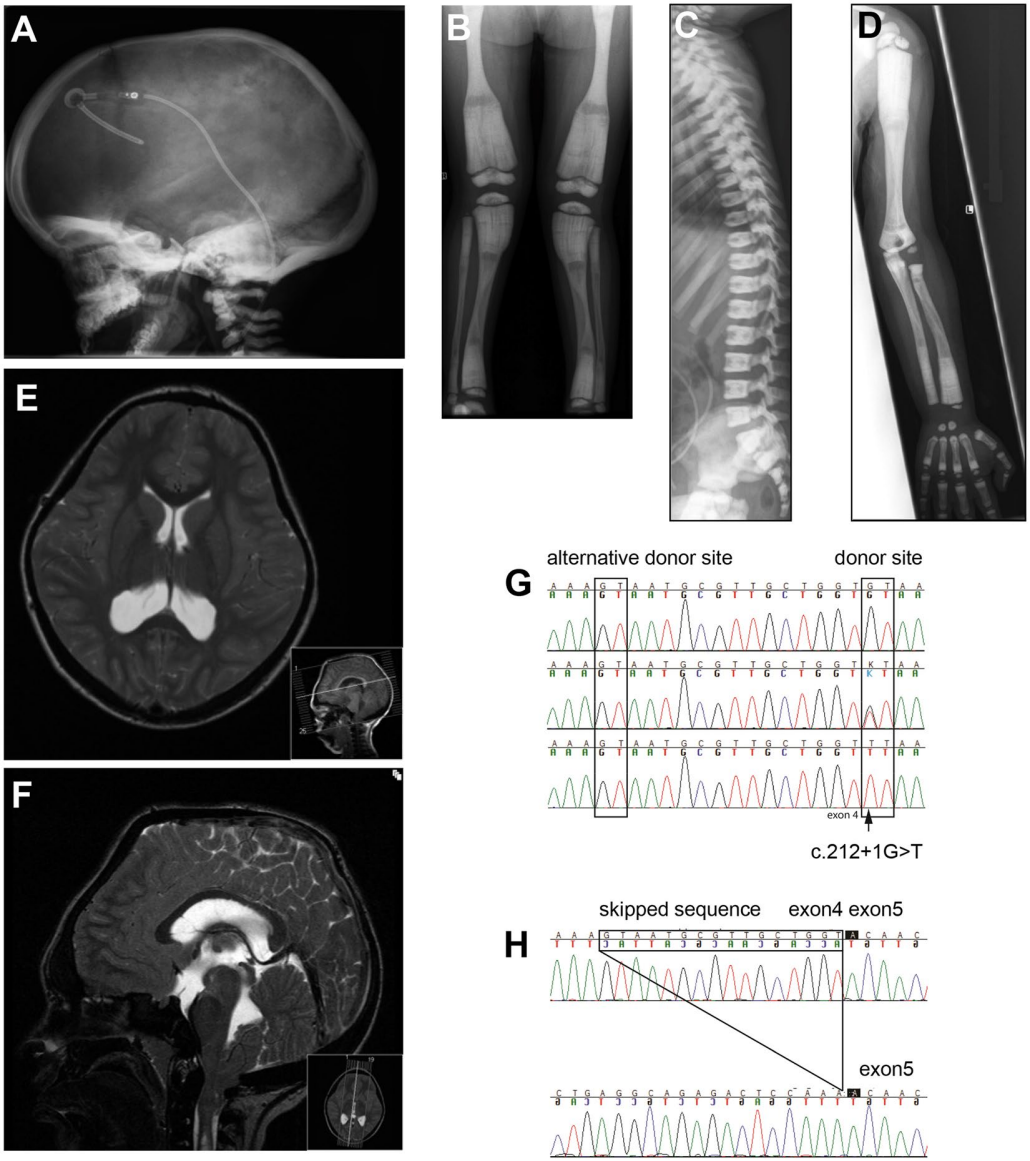
A Radiographs of subjects with the Västerbotten form of intermediate osteopetrosis, and sequencing of the *SNX10* gene. (**A**–**D**) Skeletal radiographs of a girl at the age of three years (Pt9), showing characteristic features of intermediate osteopetrosis. (**A**) Skull: Increased bone density of the skull base, frontal bossing and VP-shunt (**B**) AP view of the legs: “Erlenmeyer flask” shape of the femoral diaphysis. Longitudinal and transverse bands of lesser density in the metaphyses. Bone-within-bone pattern in the epiphyses in the knee. (**C**) Lateral view of the spine: Very dens endplates of the vertebral bodies, so called “sandwich vertebrae”. The ribs (seen in part) are broad and dens. (**D**) AP view of the left arm: No fractures or sign of rickets, “bone-within-bone” pattern of the phalanges. (**E**,**F**) Magnetic resonance imaging of the brain, and spine in an 11-year-old boy. MRI shows cerebellar tonsillar descent through the foramen magnum, a foramen magnum narrowing, and signs of brain stem compression with resulting syringo-hydromyelia. (**G**,**H**) Consequence of the c.212 + 1 G > T variant on (**G**) genomic level and (**H**) transcript level. (**G**) Sequencing of DNA from a control (top), a heterozygous carrier (middle) and a homozygous patient (bottom) for the c.212 + 1 G > T variant. The variant changes the donor splice site at the 5′ end of intron 4 from GT to TT. This leads to a predicted use of an alternative donor splice site 16 nucleotides upstream of intron 4. (**H**) Sequencing of cDNA from a control (top) and a patient (bottom) confirms the use of an alternative splice site and the skipping of 16 bp leading to a frame shift and a premature stop codon (p.S66Nfs * 15) at the protein level.

Manifestation of the disease was seen in early infancy in all patients, but the age at correct diagnosis varied from 7 weeks (Pt1) to 3 years (Pt9). The patient (Pt9) diagnosed at the age of 3 years was first thought to have a hereditary optic atrophy. Delayed tooth eruption prompted radiology and the diagnosis of osteopetrosis became apparent (Fig. 1A–D).

### Skeletal features, radiographs and fractures

Birth length was within ±2 SD (range 48–52 cm), and head circumference ±1 SD (range 34–35 cm). All patients had short final height (males: 132 cm, (−6.7 SD), 160 cm, (−2.5 SD), females: 149 cm, (−2.5 SD), 159 cm, (−1.1 SD) (Table 1)). In all patients, skeletal radiographs show a generalized increase in bone density with metaphyseal modelling defects, transverse bands of greater and lesser density in tubular bones, short wide femoral neck, and”bone-within-bone” pattern of the phalanges (Fig. 1A–D). There were no radiological signs of rickets in five evaluated patients (Pt1, Pt5, and Pt7–9). Seven of nine patients suffered from recurrent fractures up to about 30 times (Pt1), even after minor trauma. During childhood, almost all fractures were transverse diaphyseal fractures in the extremities without pronounced dislocation and they healed normally with periosteal callus. The adult patients (Pt1–5) also had femoral shaft fractures with prolonged healing or pseudoarthrosis despite external or internal fixation. One boy (Pt7), who received hematopoietic stem cell transplantation (HSCT), did not fracture and his growth normalized (Table 1).

### Neurological symptoms

All patients developed visual impairment before the age of one year, and five patients became blind (Pt1, Pt4, Pt6, Pt8, and Pt9). Decompression of the optic nerve was not performed in any of the patients. Retinal atrophy was not observed. All but one patient suffers or suffered from hearing impairment, and one patient (Pt6) developed a profound deafness (Table 1).

After birth, the head circumference increased rapidly in six patients. One girl (Pt9) was found to have hydrocephalus and cerebellar tonsillar herniation, requiring a ventriculo-peritoneal shunt. She was operated at the age of eight years with decompression of the brainstem, and she died unexpectedly, two years later, possibly due to compression of a postsurgical cephalocele. At that time, she was hospitalized for evaluation of severe back pain, and headache episodes. Two patients (Pt8, and Pt5) developed stenosis of the foramen magnum; one (Pt8) died at the age of 12 years due to spinal cord compression while the other (Pt5) is still alive at the age of 24 years. One boy (Pt6) showed intellectual disability in addition to early blindness and profound deafness. Facial paralysis was present in four patients (Pt1–4).

Evaluation of six magnetic resonance imaging (MRI), and three computed tomography (CT) scans showed a thickened and sclerotic bone of the skull in all but one patient (Table 2). Scans did not show brain malformations (Fig. 1E,F).

**Table 2:** Magnetic resonance imaging (MRI) and computed tomography scan (CT) of the skull and brain, in six individuals with *SNX10* related intermediate autosomal recessive osteopetrosis (IARO).

*Patient number*	1	1	1	4	5	7	8	8	9
*Gender* (*male/female*)	Male	Male	Male	Female	Female	Male	Male	Male	Female
*Age at the time of imaging* (*year*)	29	34	33	35	14	2	11	11	8
*MRI/CT-scan*	MRI	MRI	CT	CT	MRI	MRI	MRI	CT	MRI
Corpus callosum aplasia/hypoplasia	0	0	X	X	0	0	0	X	0
Sclerotic bone	1	1	1	1	1	0	1	1	1
Thickened bone	1	1	1	1	1	0	1	1	1
Ventriculomegaly	0	0	0	0	0	0	1	1	0
Remodelled inner table	1	1	1	0	1	0	0	1	0
Tonsillar herniation	1	1	X	0	1	0	1	X	1
Optic nerve sheath dilatation	0	0	0	0	1	0	1	1	0
Proptosis	1	1	1	1	0	0	1	X	1
Brain atrophy	0	0	0	0	0	0	0	0	0
Optic nerve atrophy	0	0	0	X	X	1	1	X	1
Optic canal stenosis	0	1	1	1	0	0	1	0	0
Ear fluid	1	1	X	X	0	1	0	X	0
Dural venous sinus stenosis	X	X	X	0	1	X	1	X	1
Foramen magnum stenosis	0	0	X	X	1	0	1	X	1
Posteriorly angulated dens with stenosis	X	X	X	X	1	0	1	X	1
Cervical spinal stenosis in included upper part	1	1	0	0	0	0	0	0	0
Subdural hematoma or intra-cerebral bleeding	0	0	0	0	0	0	0	0	0
Prominent collateral veins between sinus transversus and extra-cranial veins	0	0	0	0	0	0	1	0	0
Lemon shape of the scull (similar to what is seen in Chari II malformation), syrinx of the cervical cord, large occipital horns	0	0	0	0	0	0	1	0	0
Calcification in basal ganglia and subcortical frontal lobes	0	0	0	0	0	0	0	1	0

1 = present, 0 = not present, X = feature not possible to evaluate.

### Hematological symptoms

Anemia was present early in life in four patients (Pt1, Pt2, Pt7, and Pt9) due to bone marrow encroachment. Consumption and hemolysis of erythrocytes, followed by a low leucocyte count and thrombocytopenia, was seen in two patients (Pt8, and Pt9); haptoglobin and carboxyhemoglobin (CoHb) were below the detection limit. Splenectomies were performed on these patients and the hematopoiesis almost normalized afterwards. Six of nine patients require or required repeated blood transfusions due to normocytic anemia (Table 1).

All patients were offered stem cell transplantation, most of them during adolescence, but HSCT was accepted only by the parents of the youngest one (Pt7). He received HSCT at the age of 33 months from an HLA-matched unrelated donor; he is still alive and healthy at the age of 15 years. However, he suffers from irreversible tissue damage that occurred prior to transplantation: he has visual deterioration as well as missing and malformed teeth.

Two patients (Pt4, and Pt6) died due to a combination of pancytopenia and septicemia; one (Pt6) died at the age of 19, due to septicemia originating from osteomyelitis of the jaw, and the other (Pt4) died at the age of 35, due to chronic osteomyelitis of one femur after an unhealed fracture. Autopsy was not performed in any of the patients (Table 1).

### Symptoms of the airways, jaw and teeth

All patients had narrow nasal, and pharyngeal cavities, and two patients (Pt2, and Pt9) had documented nasal congestion before the age of one year. Three patients (Pt1, Pt8, and Pt9) needed a tracheostomy at the age of 8, 9 and 22 years, respectively. Three adult patients (Pt1–3) are treated with continuous positive airway pressure (CPAP) during nighttime, due to sleep apnea syndrome. Tooth eruption was delayed in three patients (Pt7–9), and teeth are congenitally missing, malformed or retained in all patients. Severe osteomyelitis and osteonecrosis of the jaw is seen in all adult patients (Pt1–6), with abscess formations and fistulas to the orbits and skin. One patient (Pt1) presented with osteonecrosis of the jaw at the age of six years, other patients (Pt2, Pt3, Pt5, and Pt6) at the age of 12, 13, 18 and 30 years, respectively. Osteonecrosis of the ear canal is seen in three of the eldest patients (Pt1–3) (Table 1).

### Exome sequencing, data filtering, DNA and cDNA sequencing, carrier frequency

Exome sequencing of three patients (Pt1, Pt2, and Pt5) revealed 115,283 unique SNPs. Among those, 25,301 were common, 24,867 remained after excluding SNPs on chromosomes X and Y, and 9,401 were homozygous. Nineteen of those were not present in the control population and three were non-synonymous, and located in three different genes. One of the three genes was the *SNX10* gene. Homozygous sequence variants at the donor splice site of exon 4 (c.212 + 1 G > T) were confirmed in all affected patients using Sanger sequencing, and the parents were found to be heterozygous for the splice site mutation (Fig. 1G–H). The splice site variant is not present in any of >1000 exomes present in our local Canvas DB, or in controls in Exome Seq. project or 1000 Genomes^13, 14^. Sequence analysis of cDNA from cultured patient peripheral blood cells revealed that an alternative splice site, located 16 bases upstream from intron 4, was used, resulting in a frame shift and a stop codon (p.S66Nfs * 15). The carrier frequency of the sequence variant in the county of Västerbotten is 1:93 or 1.1% of the population.

### Genealogy and haplotype analysis

Genealogic data revealed a common ancestor 8–12 generations back, in the early 19^th^ century. He was born 1820 in Bureå, Skellefteå, County of Västerbotten, northern Sweden. Haplotype analysis was performed to evaluate the age of the sequence variant. The size of the homozygous regions in Pts1–5 is 4044451 bp, 3494094 bp, 3515023 bp, 5632740 bp, 4628862 bp, and 4628862 bp, respectively. Pt9 showed homozygosity between marker rs477644 and rs6462058, corresponding to 11307603 bp, which was included in the age calculations (Fig. S1). The linkage disequilibrium in six ARO probands extends over a mean homozygous region of 5.3 cM, which equals 38 meioses/generations. With an assumed generation time of 25 years, this corresponds to a shared ancestral origin of the *SNX10* gene variant 950 years ago^15^.

### Monocytes from patients form large osteoclasts *in vitro*

We isolated CD14^+^ monocytes from the peripheral blood of three patients, (Pt1, Pt2 and Pt5) and seven healthy controls, designated C1–C7. We assessed osteoclastogenesis on both plastic and bone slices, by incubating CD14^+^ monocytes with RANKL. Cells from patients were tested alongside control cells in six different independent experiments. The data are presented separately for these individual experiments instead of being pooled because of the large inter-individual variability that is commonly seen in primary osteoclast cultures from humans.

**Figure 2:**
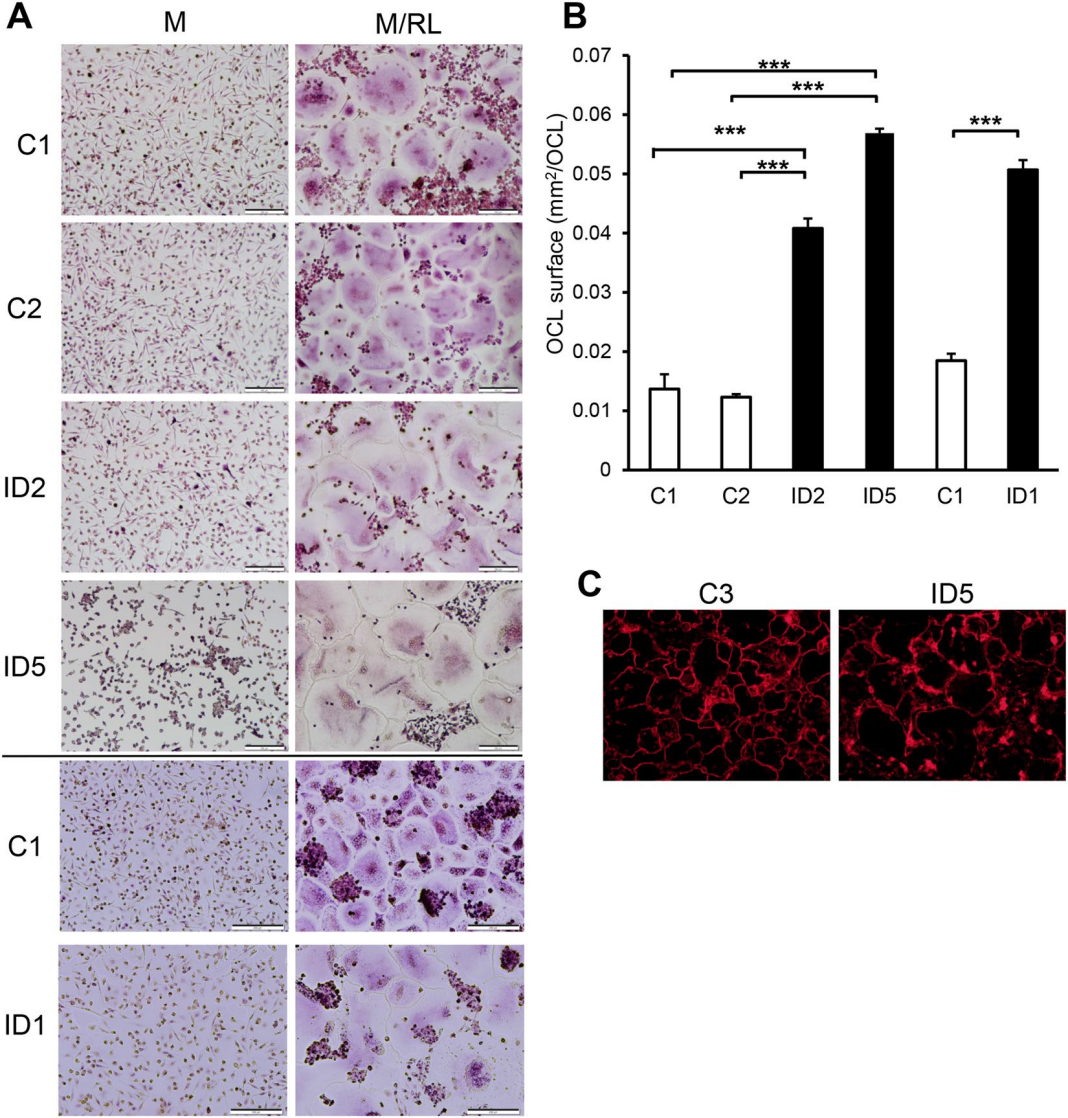
Osteoclast differentiation of CD14 ^+^ monocytes from peripheral blood. Photographs of TRAP staining (**A**) and quantification of surface area (**B**) of CD14^ +^ cells cultured in M-CSF (M) or M-CSF and RANKL (M/RL) for four days (C1, C2, Pt2, Pt5) or five days (C1, lower photos, and Pt1). Surfaces are presented as mean ± SEM. (**C**) Actin ring staining of cells cultured in M/RL for four days. Scale bars: 100 μm. *** P ≤ 0.001.

Multinucleated cells that stained positive for tartrate resistant acid phosphatase (TRAP^+^) were formed on plastic dishes in all cultures from patients and controls. The TRAP^+^ multinucleated cells (TRAP^+^MuOCL) from patients were larger and stained less intensely for TRAP than those from controls (Fig. 2A,B). Accurate counting of TRAP^+^MuOCL in patient cultures was difficult as osteoclasts in patient cultures were large and pale.

The patient TRAP^+^MuOCL formed peripheral belts of F-actin-rich podosomes similar to controls. The circumference of these actin belts was greater in the patient osteoclasts than the control osteoclasts, reflecting the greater size of the patient cells (Fig. 2C).

### Expression of osteoclastic and osteoclastogenic genes in cells from patients

**Figure 3:**
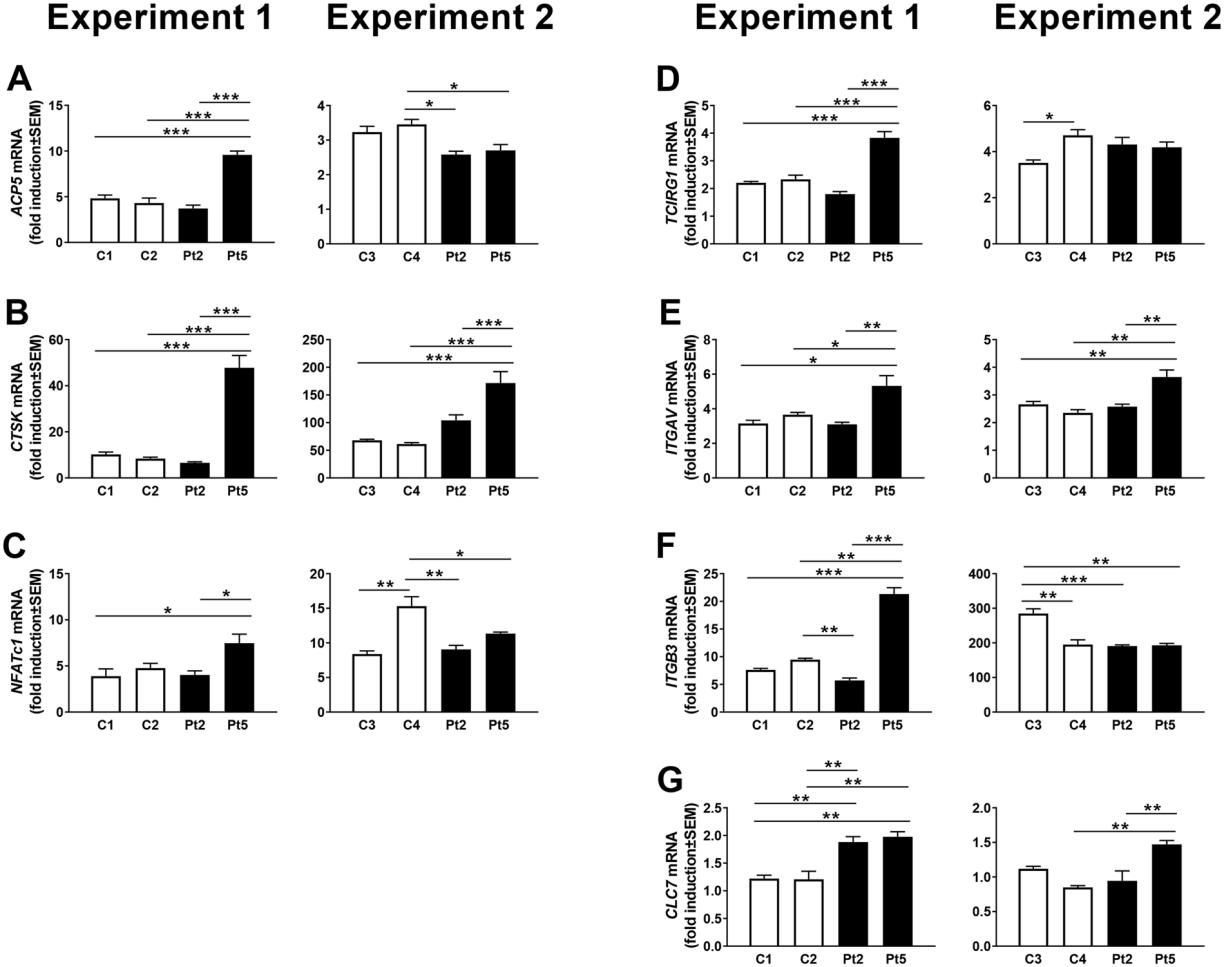
Gene expression in osteoclasts from patients (Pt2, Pt5), and controls (C1, C2, C3, C4). Fold induction of gene expression between cells treated with M-CSF (M) with and without RANKL (RL) for three days. All values are given as mean ± SEM (n = 4). *P ≤ 0.05, **P ≤ 0.005, ***P ≤ 0.001 between indicated groups.

We next assessed if the expression of genes associated with osteoclast differentiation and function was altered in the patient osteoclasts. Gene expression analyses were performed twice with cells from two patients (Pt2 and Pt5) at one time point during the culture, and with two different controls (C1–C4) in each analysis (Figs 3 and S2). The reason data are provided separately for different controls and different patients is that the logistics of the cell collections and experiments did not allow us to compare the same control with the same patient in each experiment. We performed separate experiments comparing cells from different controls and from different patients. We repeated the experiments at different times to ensure reproducibility of our observations.

The mRNA expression of *ACP5* (encoding the enzyme TRAP) and *CTSK* (encoding cathepsin K), both robust markers of the osteoclastic phenotype, was strongly upregulated by RANKL in cells from both patients and controls (Fig. 3A,B). There was no difference between patients and controls in the degree of upregulation of *NFATc1* (encoding nuclear factor of activated T-cells, the master osteoclastogenic transcription factor), (Fig. 3C). No differences between patients and controls were found in RANKL-induced upregulation of *TCIRG1* (encoding the proton pump subunit, Atp6i, essential in acidification process) (Fig. 3D), or the mRNA expression of *ITGAV* and *ITGB3*, (encoding the α_v_ and β_3_ subunits of the vitronectin receptor, essential for osteoclast attachment to bone) (Fig. 3E,F). The expression of *CLCN7* (encoding chloride channel 7, with an essential role in acidification) was consistent throughout osteoclastogenesis in cells from both patients and controls (Fig. 3G). Although some genes showed a significant difference between patient and controls in one of the two independent experiments, none of the analyzed genes were significantly and reproducibly changed in both patients compared to controls. These data indicate that the *SNX10* mutation does not affect the expression of key genes associated with osteoclast differentiation and function.

**Figure 4:**
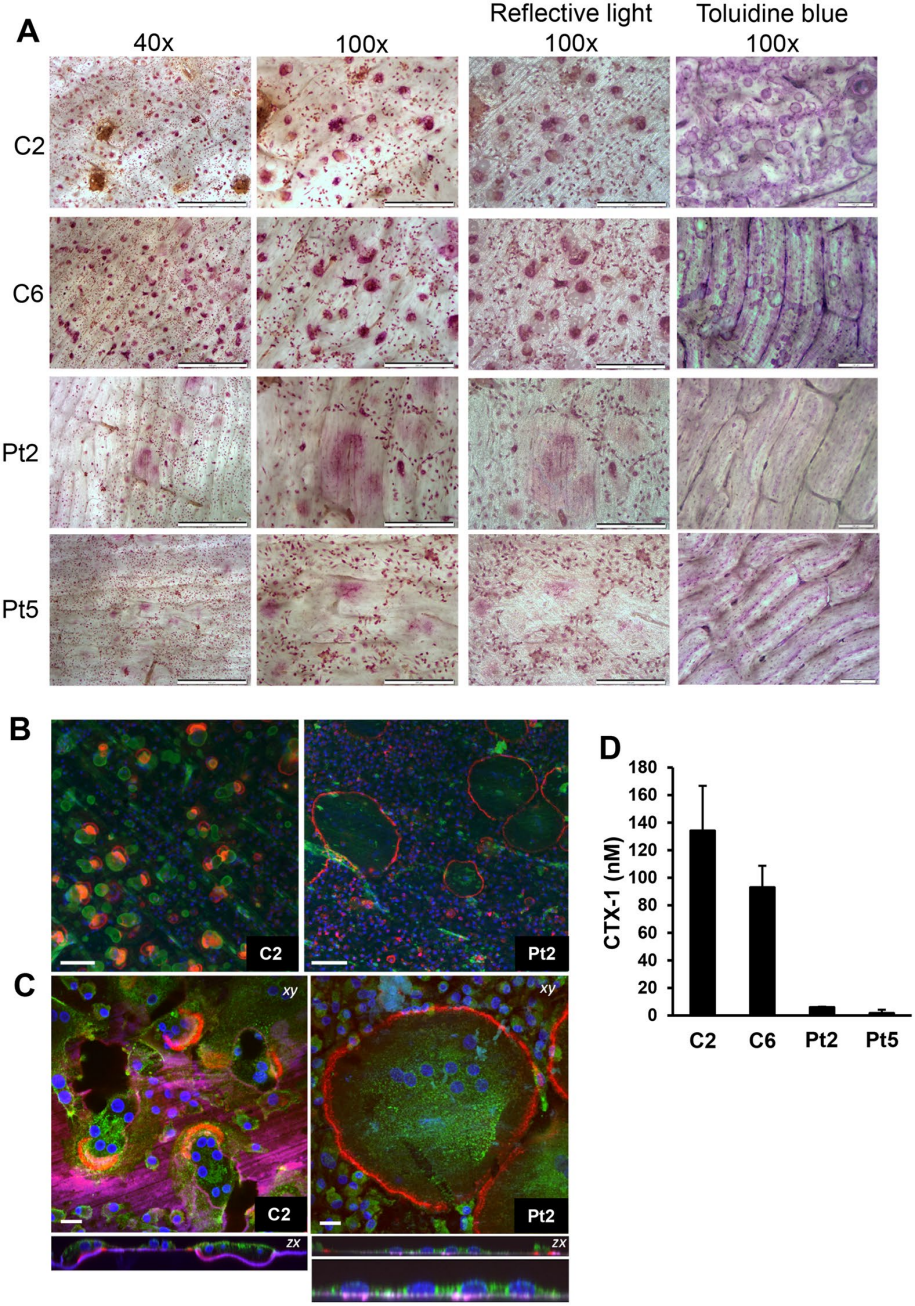
Patient-derived osteoclasts form actin rings but are unable to resorb bone. (**A**) TRAP staining of CD14 ^+^ cells cultured in M/RL on bone for eight days. Scale bar 40× panel: 500 μm, scale bar 100× light microscope and reflective light panel: 200 μm, scale bar 100× toluidine blue panel: 100 μm. (**B**,**C**) Osteoclasts were analyzed by confocal microscopy; (**B**) stained with TRITC-phalloidin (red), DAPI (blue) and the bone surface using a fluorescent bisphosphonate (AF-ALN; green). Resorption pits stain brightly with AF-ALN and can be seen in the control only. Scale bar = 100 μm; (**C**) stained with TRITC-phalloidin (red), DAPI (blue) AF-ALN (magenta) and the cell membranes with wheat germ agglutinin (green). AF-ALN omitted from upper panel of Pt2 for clarity. Black patches in C2 xy image indicates resorption pits. Bottom panel of Pt2 is a zoom of the *zx* section illustrating lack of resorption. Scale bar = 20 μm; (**D**) CTX-1 release in media during day 6 to 8 of culture in M/RL. C1, C6 = controls, Pt2, Pt5 = patients (n = 4–5).

The ability of osteoclasts with the *SNX10* mutation to resorb bone was analysed by culturing CD14^+^ cells from patients on slices of devitalized bovine bone in six different experiments, each comparing cells from one of 3 patients with several of 7 controls. In control cultures, RANKL stimulated the formation of many TRAP^+^MuOCL. In patient cultures we observed TRAP^+^MuOCL, but, as in cultures on plastic, we also noticed several much larger cells with weak TRAP staining, making accurate counting of the number of osteoclasts impossible (Fig. 4A). We, therefore, also used TRAP5b released into the culture media as a parameter of mature osteoclast formation^16^, but found no consistent difference between patients and controls (data not shown).

Phalloidin staining demonstrated that control osteoclasts frequently exhibited crescent-shaped actin rings, which were associated with extensive resorption pits and resorption trails in the bone slices (Fig. 4B,C) and release of CTX (a degradation fragment from collagen type I) (Fig. 4D). Patient osteoclasts polarized their actin cytoskeleton in rings at the periphery of the cells, similar to the actin belts seen in cells on plastic. Patient cultures, however, showed no evidence of resorption. There were no resorption pits (Fig. 4A–C) and there was no release of CTX (Fig. 4D). There was no evidence of vacuolation in any of the patient osteoclasts (Fig. 4B).

### Increased size of patient osteoclasts is independent of precursor fusion

Individual patient osteoclasts occupied larger areas of the bone surface than control osteoclasts (Fig. 4A–C), similar to the observations on plastic (Fig. 2A–C). However, osteoclasts on bone from patients contained similar numbers of nuclei as those from healthy controls (mean nuclei/osteoclast = 7.3 for C1, 7.8 for C2, 7.9 for Pt2). Patient osteoclasts were much flatter than the control osteoclasts; in cross-sectional views most osteoclasts were no deeper than a single nucleus (Fig. 4C). This indicates that the larger size of the patient osteoclasts is the result of increased spreading, rather than the result of increased fusion.

In agreement with these findings, we were unable to observe any significant differences replicated in two separate experiments between expressions of several genes suggested to be involved in osteoclast fusion (Fig. S2). RANKL-induced upregulation of the osteoclast fusion genes *DC-STAMP* and *OC-STAMP* ^17–19^ was similar in cells from patients and controls (Fig. S2). Syncytin-1 and its receptor Asct2 have also been suggested to be involved in osteoclast fusion^20^ but the mRNA expression of the genes encoding these proteins (*ERVW-1* and *SLC1A5*, respectively) was not different between patients and controls (Fig. S2).

### Osteoclasts exhibiting *SNX10* mutation have an impaired ruffled border

**Figure 5:**
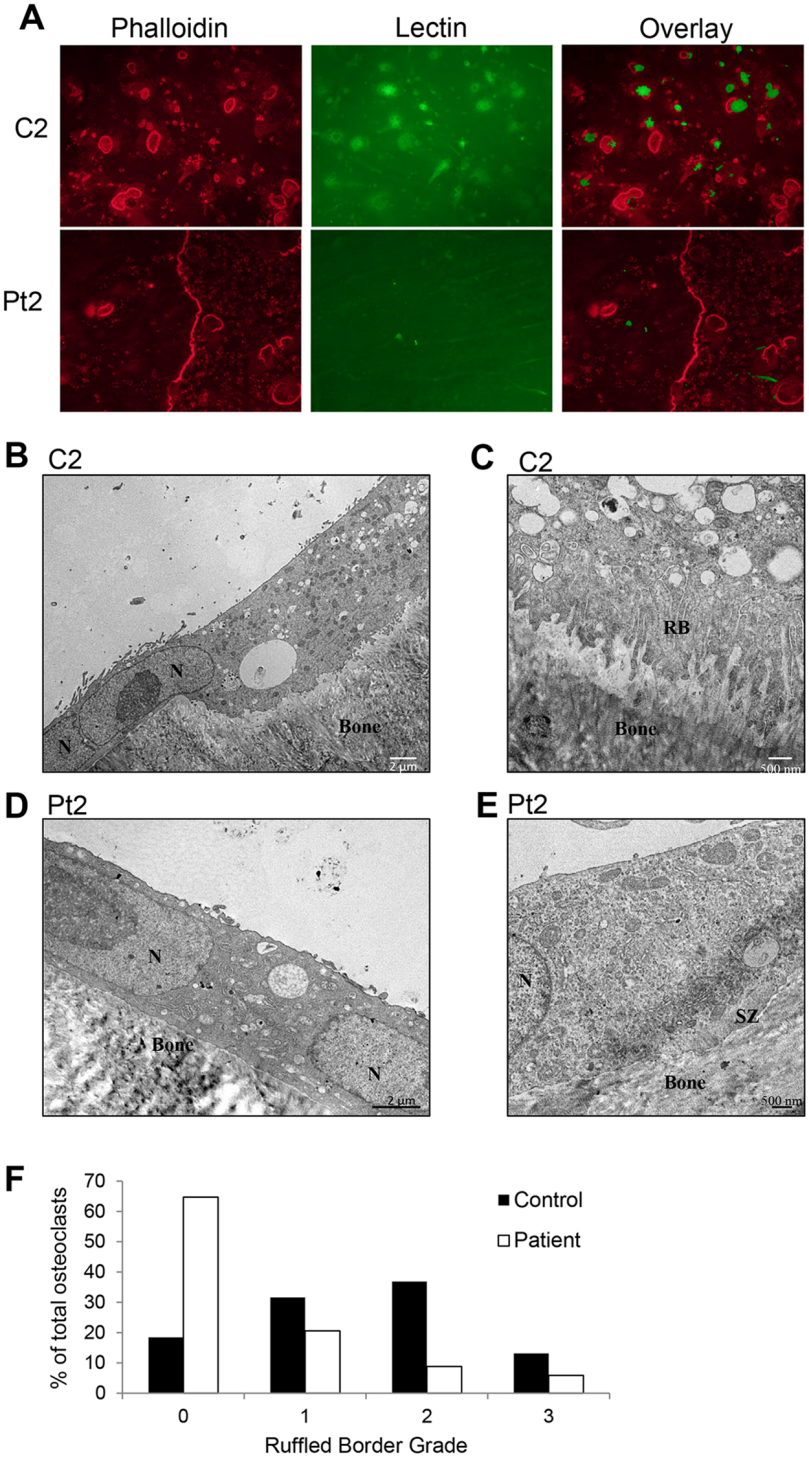
Patient-derived osteoclasts formed defective ruffled borders. (**A**) Phalloidin staining for acting rings (left) and lectin staining for ruffled border (middle) of CD14^ +^ cells cultured in M/RL on bone for 11 days. (**B**–**E**)-Representative TEM micrographs of control C2 (**B**,**C**) and patient Pt2 (**D**,**E**) -derived osteoclasts show extensive ruffled border (grade 3) and resorption in the control, compared to lack of ruffled border (grade 0) and resorption in the patient. Patient osteoclasts formed sealing zones (**E**). N = nucleus, SZ = sealing zone, RB = ruffled border. Scale bars in left panels = 2 μm. Scale bars in right panels = 500 nm. (**F**) The percentage of osteoclasts categorized into each ruffled border grade (control, n = 38; patient, n = 34).

Since osteoclast formation and F-actin ring formation was unaffected, we speculated that the lack of resorption by osteoclasts derived from the patients would be due to defective ruffled border formation, similar to well-characterized cases of osteoclast-rich ARO^21^. This would be consistent with the likely role of SNX10 in vesicular trafficking processes in osteoclasts. While control osteoclasts on bone accumulated PNA-lectin (a marker of the ruffled border)^22, 23^ within the actin ring, patient osteoclasts showed no PNA-lectin staining (Fig. 5A), suggesting impaired ruffled border formation in the patient osteoclasts.

Transmission electron microscopy was used to further examine the sealing zone (F-actin rings) and the ruffled border in cultured osteoclasts. This ultrastructural analysis confirmed that patient osteoclasts retained the capacity to form sealing zones (Fig. 5E), but that 65% of patient osteoclasts lacked ruffled borders (Grade 0) and showed no evidence of bone resorption. In contrast, control osteoclasts showed evidence of extensive bone resorption and only 18% of these cells lacked ruffled border (Fig. 5B,D and F). Fifteen percent of patient osteoclasts showed moderate/extensive ruffled borders, compared to 50% of control osteoclasts. The ultrastructural analysis confirmed that patient osteoclasts were very large and flat compared to control cells, with sealing zones located at the cell periphery.

### Gene expression of wild type and mutated *SNX10*

**Figure 6:**
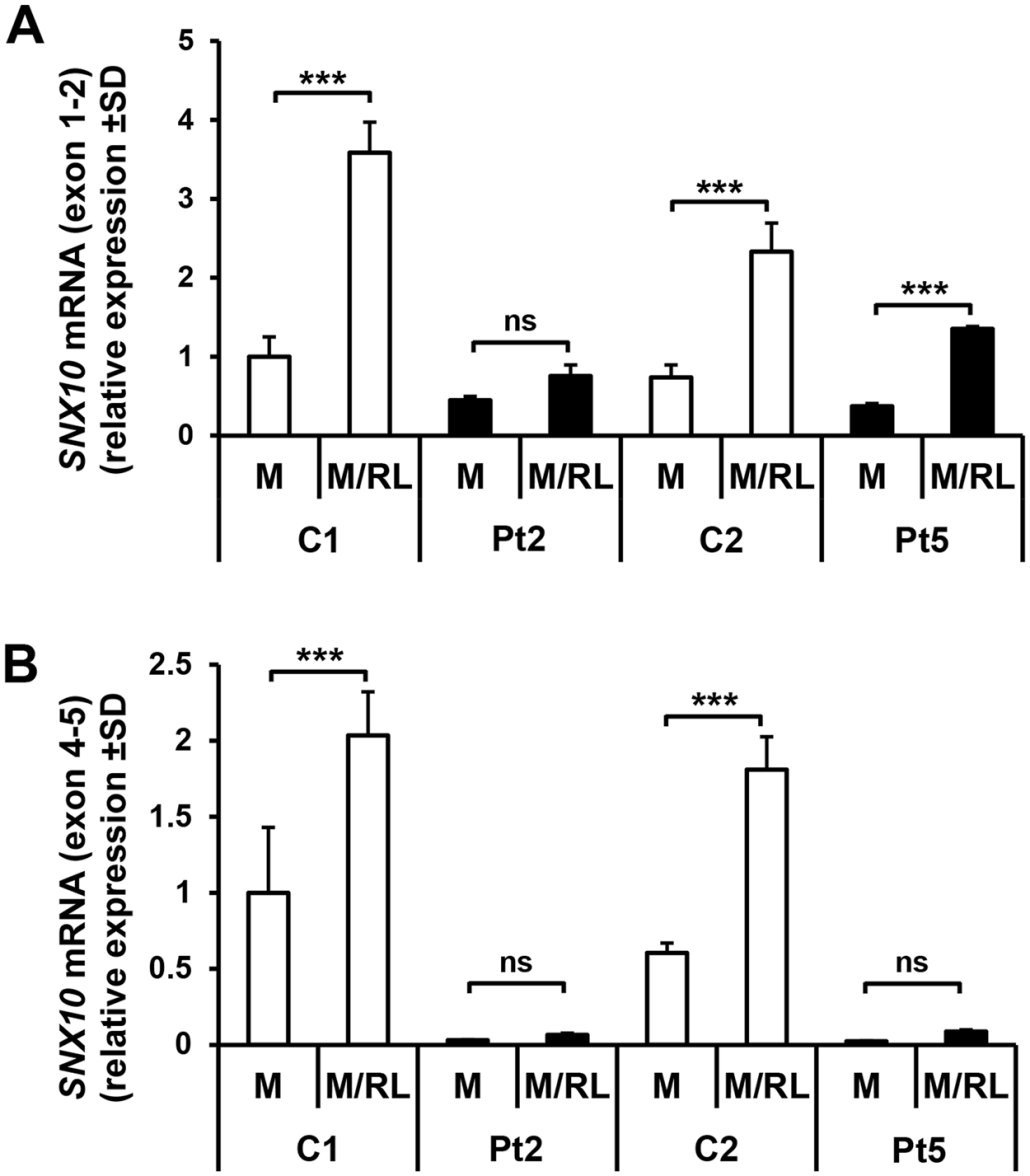
Expression of *SNX10* mRNA in cells treated with M or M/RL for three days, analyzed with an assay covering the exon 1–2 (**A**) and exon 4–5 (**B**) junction. Values are given as mean ± SD (n = 2–4). ***P ≤ 0.001 between M and M/RL treated cells.

*SNX10* mRNA levels were measured in RANKL-stimulated CD14^+^ cells using two different TaqMan assays, spanning the junction between exon 1–2 and 4–5, respectively. *SNX10* mRNA, analyzed with the exon 1–2 assay, was present in patients and controls and significantly upregulated by RANKL in controls and in one of the two patients (Fig. 6A). However, the mRNA levels were lower in patient cells. The assay spanning the exon 4–5 junction also showed upregulation of *SNX10* by RANKL in control cells (Fig. 6B). However, as the mutation is located at the splice donor site of exon 4, this domain was barely detected in cells from patients.

## Discussion

We herein report the clinical features and natural course of the Västerbotten form of IARO [MIM 615085], the disease-causing mutation, its origin and age. We also present functional analyses of the effects of this mutation on osteoclast differentiation and function.

The Västerbotten form of IARO is caused by a splice site mutation in the *SNX10* gene [MIM 614780] c.212 + 1 G > T^12^ resulting in a frame shift and a stop codon (p.S66Nfs * 15). The carrier frequency was found to be 1/93 in the population of Västerbotten. According to haplotype analysis, the shared ancestral origin of the *SNX10* gene variant is approximately 950 years old.

The phenotype of IARO, previously associated with mutations in the *PLEKHM1*, *CLCN7*, and *CA2* genes^1–3, 21, 24^, is defined as asymptomatic at birth, with patients exhibiting fractures by the end of their first decade of life, while bone marrow failure and hepato-splenomegaly are rare^24–27^. In contrast, our data show that patients with *SNX10-*related IARO of Västerbotten type described here have a more severe phenotype, with age at onset in early infancy, presenting with optic atrophy, macrocephaly, frontal bossing, proptosis, and anemia. Splenomegaly, due to compensatory extra-medullary hematopoiesis, was present before the age of five, and splenectomy was performed in three patients (Pt1, Pt8, and Pt9), the youngest at the age of five years (Pt1). Although the patients displayed many of the symptoms seen in malignant ARO, they all survived early childhood without being transplanted. We, therefore, placed the patients in the group of IARO, rather than in the group of malignant ARO in which patients do not survive childhood if not transplanted.

Osteopetrosis presenting with hydrocephalus, hind-brain posterior fossa crowding, foramen magnum narrowing, and syringohydromyelia is rare, but has been described in the literature^28–31^. Cure *et al*. studied cranial MRI of osteopetrosis, but did not identify ventriculomegaly, tonsillar herniation, or remodelled inner table in IARO^32^. In the Västerbotten intermediate form of osteopetrosis these features seem to be common; in our study, four patients (Pt1, Pt5, Pt8, and Pt9) had cerebellar tonsillar descent through the foramen magnum, and three (Pt1, Pt5, and Pt8) of them showed remodelled inner table. Three subjects (Pt5, Pt8, and Pt9) had foramen magnum stenosis, posteriorly angulated dens with stenosis and signs of brain stem compression.

Structural brain malformation is rarely reported as part of the clinical spectrum of osteopetrosis, but cerebral atrophy, corpus callosum agenesis, hypoplasia of the hippocampus, and Dandy-Walker malformation has been reported^33–36^. However, none of our *SNX10* related IARO patients had intra-cerebral malformations. MRI of the brain showed calcification of the basal ganglia, and subcortical frontal lobes in one male patient (Pt8), who died due to spinal cord compression caused by a combination of prominent calvarial and skull base thickening, toncillar herniation, foramen magnum narrowing and posteriorly angulated dens with stenosis. He also had ventriculomegaly, dural venous sinus stenosis, and remodelled inner table as a sign of elevated intra-cranial pressure. MRI also showed collateral veins between sinus transversus and extra-cranial veins, possibly caused by a jugular foramen stenosis, occlusion of the jugular vein and thrombosis of the transverse and sagittal sinuses, which is not previously described in subjects with IARO. However, no brain phenotype was reported in *Snx10* knock out mice^37^. Calcification of basal ganglia, dura mater, tentorium cerebelli and periventricular calcification extending into the white matter (Marble brain disorder) has previously been described with or without association to renal tubular acidosis due to carbonic anhydrase II deficiency^38–41^. The reason for calcification of the brain is unclear.

Results of studies performed in *Snx10* knock out mice suggest an additional role for *Snx10* in the gastric epithelium. *Snx10* knock out mice have impaired gastric acidification and are severely hypocalcaemic compared to wild type littermates, resulting in rickets as well as osteopetrosis (osteopetrorickets). In contrast, osteoclast-specific knockout of *Snx10* resulted in osteopetrosis without rickets or alterations in calcium balance^37^. Interestingly, the patients in the present study have no signs of rickets. Also, *SNX10* osteopetrotic patients have been shown to be almost completely rescued from the sclerotic phenotype by hematopoietic stem cell transplantation^12^. Considering the results of these studies, there seems to be a difference in the importance of *SNX10* in different organs in mice and humans.

Using RANKL-stimulated peripheral blood monocytes we found that osteoclasts could be generated from cells isolated from the patients bearing the *SNX10* splice site mutation. This is consistent with previous studies, which were also able to generate osteoclasts from peripheral blood of patients with different *SNX10* mutations^10^, and from spleen or bone marrow cells of *Snx10* KO mice^37, 43^. In contrast, silencing of *Snx10* in the mouse pre-osteoclastic cell line Raw 264.7 was shown to inhibit RANKL-induced osteoclast differentiation^42^. Moreover, *SNX10* mRNA levels were increased during RANKL-stimulated osteoclastogenesis, consistent with a likely role in osteoclast function rather than formation. Although we have not been able to obtain bone biopsies from the patients, these findings suggest that the patients have an osteoclast-rich form of osteopetrosis.

The osteoclasts generated from patients had normal expression of key osteoclast genes including those associated with osteoclast fusion and bone attachment, however, the osteoclasts were larger than in healthy controls. There was not difference in the number of nuclei per osteoclasts between patients and controls, indicating that the larger size is the result of increased spreading, similar to what is seen in other cases of IARO^21^. By contrast, Aker *et al*. reported that osteoclasts generated from patients with a missense mutation (Arg51Gln) in *SNX10* were fewer in number and smaller than in controls and, similar to osteoclasts from the *Snx10* null mouse, had a markedly decreased capacity to dissolve calcium phosphate^10, 37, 43^. Our data show that osteoclasts cultured from patients were unable to resorb bone, evidenced by both an absence of resorption pits and lack of CTX in the culture supernatant. This finding is identical to earlier findings with osteoclasts cultured from IARO patients bearing mutations in *PLEKHM1* ^21^. Interestingly, human osteoclasts with a missense *SNX10* mutation^10^, and mouse osteoclasts from *Snx10* null mice^37, 43^ although having reduced activity, still had some capacity to dissolve calcium phosphate film, whereas human osteoclasts from our patients did not show any evidence of bone resorbing activity. This discrepancy most likely is due to the use of crystalline calcium phosphate coating instead of bone as substrate for the assessments of the bone resorbing activity.

Electron microscopy revealed that the inability of osteoclasts from osteopetrotic patients in the Västerbotten County to resorb bone is associated with defective ruffled border formation, very similar to patients with ARO caused by mutations in other genes^4, 21, 44^. Using phalloidin staining, we found that the SNX10 patient osteoclasts were still able to form F-actin rich podosome belts in plastic dishes and normal F-actin rings when cultured on bone. In agreement with these observations, electron microscopy demonstrated these osteoclasts formed a sealing zones with normal appearance. These observations indicate that the defect lies in the endosomal/lysosomal trafficking pathways associated with ruffled border formation. This is perhaps not surprising, since SNX10 is a member of the sorting nexin family of proteins that play crucial roles in cargo sorting in the endosomal pathway^45, 46^, moreover; other genetic mutations that cause osteoclast-rich ARO also affect this pathway^4^. In contrast to these findings, osteoclasts generated from bone marrow macrophages with deletion of the *Snx10* gene have been reported to form an abnormal F-actin positive podosome belt in plastic dishes^43^. We have no explanation for these differences between mouse and human osteoclasts. As discussed below, we do not believe that the difference might be due to expression of a truncated, functional form of SNX10 in our patients.

There seem to be clear differences in the functional consequences for osteoclasts in patients with the Arg51Gln mutated *SNX10* ^10^ and those in the present study. Thus, the Arg51Gln mutation results in decreased osteoclast formation and decreased osteoclast activity, whereas the *SNX10* mutation found in the Västerbotten form of IARO impairs resorption by disrupting ruffled border formation in mature osteoclasts. Furthermore, osteoclasts with the Arg51Gln mutation exhibit extensive cytoplasmic vacuolation, which was not seen in osteoclasts with the Västerbotten mutation. Increased vacuolation has also been observed by overexpressing *SNX10* in several human cell lines^47^, which raises the possibility that the Arg51Gln mutation results in gain of function; however, overexpression of the Arg51Gln form of *SNX10* does not cause vacuolation^48^, therefore, the functional consequences of this mutation remain unclear. No full length SNX10 protein can be expressed in osteoclasts with the Västerbotten mutation, as the mutation leads to a stop codon upstream from intron 4. We do not know whether a truncated form of SNX10 is expressed but we have not been able to conclusively show the presence of a truncated SNX10 protein using four different antibodies in Western blots, including antibodies recognizing the amino-terminal part of SNX10. SNX proteins contain a conserved phox-homology (PX) domain, which can bind phosphorylated phosphatidylinositols (PI), thereby targeting SNXs to PI-enriched membranes. The PX domain extends from c.30–381 in exons 3–6. It seems as if the PX domain in SNX10 is even further extended (PXe) in the C-terminal part^48, 49^. Since PX or PXe is the functional domain in SNXs, it is unlikely, even if a truncated SNX10 protein is expressed in osteoclasts carrying the Västerbotten mutation, that this truncated protein has any biological activity. The difference between the Arg51Gln mutation and the Västerbotten mutation is most likely responsible for the differences seen in osteoclast formation and function.

In conclusion, the Västerbotten intermediate form of osteopetrosis is caused by a splice site sequence variant in the *SNX10* gene and differs in clinical characteristics from other intermediate types of ARO. The age at onset is early in infancy, optic atrophy and anemia is present at diagnosis and tonsillar herniation, foramen magnum stenosis, and severe osteomyelitis/osteonecrosis of the jaw are common. Osteoclasts generated from peripheral blood monocytes of the patients showed defects in ruffled border formation and failed to resorb bone. These data extend the clinical spectrum of IARO and demonstrate a critical role for *SNX10* in ruffled border formation.

## Materials and Methods

### Subjects

We collected clinical data, medical records, x-ray, computed tomography scan (CT) and magnetic resonance imaging (MRI) of the brain, and blood samples from five male and four female patients with IARO. We also collected blood samples from their parents and siblings. Patients descended from the county of Västerbotten, northern Sweden.

### Exome sequencing

DNA from three patients and 15 non-related individuals (controls) from the county of Västerbotten were sequenced. Exome enrichment was performed using 3 μg genomic DNA. DNA samples were sheared by sonication with the Covaris S2 instrument (Covaris, Inc.). Fragment libraries were constructed from the sheared samples using the AB Library Builder System (Life Technologies) and target enrichment was performed according to the manufacturer’s protocols (Agilent SureSelect Human All Exon v3 kit). Exome capture was conducted by hybridizing the DNA libraries with biotinylated RNA baits for 24 h followed by extraction using streptavidin coated magnetic beads. Captured DNA was then amplified followed by emulsion PCR using the EZ Bead System (Life Technologies) and sequenced on the SOLiD5500xl system. Individual libraries were labelled by a post-hybridization barcoding procedure (Agilent, Santa Clara, CA, USA; barcodes compatible with SOLiD sequencing technology).

### Alignment and variant calling

Alignment of color space reads to the human reference genome (GRCh37/hg19) was performed using v2.1 of the Lifescope Software (Life Technologies). Single nucleotide variants (SNVs), and small insertions and deletions (indels) were subsequently identified by the diBayes algorithm available within the Lifescope software. All identified SNVs and indels were imported into a local installation of the CanvasDB database system^13^, for annotation and further analysis of the variants.

### DNA and cDNA sequencing, haplotype analysis, carrier frequency

DNA was extracted from whole blood using a standard salting-out method. SNX10 gene amplification and bidirectional sequencing of exons and intron-exon boundaries were performed in samples from all patients and their parents. RNA was extracted from cultured patient peripheral blood monocytes and converted to cDNA as described below for gene expression analysis. Bidirectional Sanger sequencing (Applied Biosystems BigDye Terminator v3.1 Cycle Sequencing Kit, Applied Biosystems) on a 3730xl DNA Analyzer (Applied Biosystems) was used for analysis of SNX10 cDNA (NM_001199837.1) using primer in exon 3 to 5 to confirm the mutation and to identify the effect of the splicing. Sequence analysis was performed using the Sequencher software (Gene Codes Corporation, Ann Arbor, MI, USA). Primers sequences where designed using Primer 3 Plus software (primer3plus.com/) and are available upon request. Five patients (Pt1–5) were analyzed using the GeneChip Mapping 250 K array (Affymetrix, Santa Clara, CA), and one patient (Pt9) was analyzed using the HumanOmniExpressExome-8v1array (Illumina), for haplotype analysis, and estimation of the age of the sequence variant. To calculate the carrier frequency, DNA from 1000 randomly chosen individuals from the county of Västerbotten was used (The medical bio-bank, Umeå university hospital, Umeå, Sweden)^50^.

### *In vitro* culture of CD14^+^ monocytes from patients and controls

Peripheral blood mononuclear cells were isolated by Ficoll-Paque PLUS separation and CD14^+^ cells were labelled with CD14 MicroBeads and extracted using a MACS column according to the manufacturer’s instructions (Miltenyi). Cells were then seeded in 96-well plates (3 × 10^5^ cells/cm^2^) in complete α-MEM medium (Gibco cat no 22561-021) supplemented with 10% heat inactivated fetal bovine serum (FBS, Sigma cat no F7524), 2 mM GlutaMAX (Gibco cat no 35050-038), 50 µg/ml gentamicin (Gibco cat no 15750-037), 100 U/ml penicillin and 100 µg/ml streptomycin (Gibco cat no 15140-148) and 30 ng/ml human M-CSF (M, R&D Systems cat no 216-MC-025/CF). For osteoclast generation, 2 ng/ml recombinant mouse RANKL (RL, R&D Systems cat no 462-TEC-010) was added from the start of the experiments (day 0). Media was replenished every three days and the cells were stained for TRAP at different days of culture using the Acid Phosphatase, Leukocyte (TRAP) Kit from Sigma (cat no 387A). TRAP^+^ cells containing three or more nuclei were counted as TRAP^+^ multinucleated osteoclast (MuOCL).

### Analysis of osteoclast differentiation, gene expression and bone resorption

RANKL-stimulated osteoclast differentiation of CD14^+^ cells was analyzed on plastic and on devitalized bovine bone discs (IDS Immunodiagnostics Systems cat no TDT-1BON1000-96). The surface area of TRAP positive cells on plastic were analyzed using the Osteomeasure^TM^ software (v3.2.1.0, Osteometrics Inc.). Total RNA was purified from cells at day 3 using RNeasy Micro Kit (Qiagen). cDNA synthesis was performed using the High Capacity cDNA Reverse Transcription Kit from Applied Biosystems. Gene expression was analyzed in two different experiments with in total four healthy controls and two patients using predesigned TaqMan Assays (Life Technologies) and the StepOnePlus Real-Time PCR system (Applied Biosystems). We analyzed *ACP5* (Hs00356261_m1), *CTSK* (Hs01080388_m1), *NFATc1* (Hs00542678_m1), *TCIRG1* (*ATP6i*, HS00990751_m1), *CLC7* (Hs01126462_m1), *ITGAV* (Hs00233808_m1), *ITGB3* (Hs01001469_m1), *DC-STAMP* (Hs00229255_m1), *OC-STAMP* (Hs00875776_m1), *ERVW-1* (*Syncytin-1*, Hs02341206_g1), *SLC1A5* (*Asct2*, Hs00194540_m1), *SNX10* exon 2–3 (Hs01007224_m1) and *SNX10* exon 4–5 (HS01007226_m1). The mRNA abundance of each gene was adjusted for the expression of 18S (Life Technologies 4310893E) used as an internal control. In most figures, gene expression is presented as the fold increase between M/RL and M treated cells. Statistical analysis was performed using One-Way ANOVA with Tukey’s post hoc test. The bone resorptive activity of osteoclasts on bone slices was analyzed by measuring the release of C-terminal telopeptides of type I collagen (CTX) in culture supernatants using a commercial ELISA (IDS Immunodiagnostics Systems cat no AC-07F1) and by Toluidine blue staining of positive excavations. TRAP5b was analyzed in culture media using commercial ELISA (IDS Immunodiagnostics Systems cat no SB-TR201A).

### Staining for the actin ring and ruffled border

Actin organization was studied at day 4 on plastic and day 11 on bone. Cells were fixed in 4% paraformaldehyde, washed with PBS and then permeabilised using 0.1% Triton X-100 in PBS for 10 min at 4 °C. After washing again with PBS, the cells were stained with rhodamine conjugated phalloidin (Life Technologies cat no R415), 5 U/ml in 2% BSA/PBS, for 20 min at 4 °C. Following washing in PBS the cells were mounted in Prolong Gold Mountant (Life Technologies cat no P-36931). Cells cultured on bone were stained for phalloidin as above but including 100 µg/ml FITC conjugated peanut agglutinin (PNA)-lectin (Sigma cat no L7381) to stain the ruffled border^22, 23^. Photographs were taken using widefield fluorescence microscopy.

### Confocal microscopy

Osteoclasts cultured on bovine bone discs were prepared as above, and, in addition to phalloidin, were stained with 5 μg/ml Alexa Fluor 633-conjugated wheat germ agglutinin to stain cell membranes and 10 μM Alexa Fluor 488-conjugated alendronate (AF-ALN, ref. 51) to stain bone surfaces. Bone discs were mounted on to glass slides in VectaShield with DAPI (to stain nuclei) mounting medium before analyzing on a Zeiss LSM700 confocal microscope. To assess potential differences in fusion, the number of nuclei was counted in 80 randomly selected osteoclasts from two control cultures and one patient culture.

### Transmission Electron Microscopy investigation of ruffled border morphology

To investigate whether patient osteoclasts were capable of forming mature ruffled borders, cells cultured on bovine bone were analyzed by transmission electron microscopy (TEM). Briefly, cell cultured with M-CSF and RANKL for eight days were fixed in 2.5% glutaraldehyde in 0.1 M cacodylate buffer. The bone discs were then demineralized in 0.15 M EDTA before being postfixed in 1% osmium tetroxide and embedded in Epon resin. Bone discs were sectioned perpendicular to the bone surface to obtain cross sections of osteoclasts and their ruffled border areas. Ultrathin sections were imaged using a JEOL Jem-1400Plus TEM equipped with an AMT UltraVUE camera. Following image acquisition, osteoclasts were graded based on their ruffled border morphological characteristics, similarly to a previous study, ref. 52: grade 0 - no ruffled border apparent; grade 1 - rudimentary ruffled border, with few poorly-developed membrane folds apparent; grade 2 - moderate ruffled border, with folded membrane apparent at some areas; grade 3 - extensive, highly-folded ruffled border.

### Statistics

For experiments with two patients and two controls, statistical analysis was performed using One-Way ANOVA with Tukey’s post hoc test. When one patient was compared to one control, a two-sided Student’s t test was used. A difference was considered statistically significant with *P* < 0.05. Due to the limited number of patients and the large inter-patient variation, it was not possible to pool the results and make statistical comparisons based on the patients as one group. Instead, the results are shown for each patient separately. For gene expression analyses (Figs 3 and S1) each independent experiment is shown.

### Study approval

The Regional Ethical Review Board at Umeå University, Umeå, Sweden, approved the study. Patients, parents or first-degree relatives gave their written informed consent. Methods using human tissue were in accordance with the Helsinki declaration.

## Electronic supplementary material

Supplementary Figure S1 and S2
