# Supplementary material for: SNX10 gene mutation leading to osteopetrosis with dysfunctional osteoclasts
Source: Sci Rep. 2017 Jun 7;7:3012. doi: 10.1038/s41598-017-02533-2 (PMC5462793; doi:10.1038/s41598-017-02533-2)
Supplement: Supplementary file 1 — Supplementary Figure S1 and S2 [file 41598_2017_2533_MOESM1_ESM.pdf]

		1	2	3	4	5	6			
		Pt 4	Pt 2	Pt 3	Pt 1	Pt 5	Pt 9			
**dbSNP RS ID**	**Physical Position**	**Call**	**Call**	**Call**	**Call**	**Call**	**Call**			
rs1583511	22726162	AA	AA	AA	AB	AB				
rs722293	23488693	AB	AA	BB	AA	AA				
rs104987	23840001	AB	AB	AB	BB	AB				
rs725237	23840172	BB	BB	BB	BB	BB				
rs156318	23955628	NoCall	BB	NoCall	NoCall	BB				
rs16091	24089998	NoCall	AB	NoCall	BB	BB				
rs2106709	24156719	BB	AB	BB	BB	BB				
rs2188880	24538029	AA	AA	NoCall	AA	AA				
rs763852	24704471	BB	BB	BB	BB	BB				
rs6461849	24971325	AA	AA	AA	AA	AA				
rs6461850	24971658	AA	AA	AA	AA	AA				
rs721317	25095041	BB	BB	BB	BB	BB				
rs721316	25095081	AB	BB	BB	BB	BB				
rs731008	25302784	BB	BB	BB	BB	BB				
	25302977	AB	AA	AA	AA	AA		Minimal region		
SNX10 c.212+1		BB	BB	BB	BB	BB		2052047		
rs986706	27355024	BB	BB	AB	BB	AB				
rs723699	27371748	BB	BB	BB	BB	BB				
rs757397	27650813	AA	AB	AA	AA	AA				
rs735664	27651309	BB	BB	AB	BB	AB				
rs720659	27658490	BB	BB	AB	BB	AB				
rs3886550	27804544	AA	AA	AA	AA	AA				
rs917117	27918577	BB	NoCall	NoCall	BB	NoCall				
rs1073297	28107669	BB	NoCall	NoCall	NoCall	BB				
rs1073298	28107747	BB	BB	BB	BB	BB				
rs65264	28287883	NoCall	BB	BB	NoCall	NoCall				
rs150613	28358902	AA	AA	AA	AB	AB				
rs763809	28633529	BB	BB	BB	BB	AB				
rs717891	28742646	AA	AB	AB	BB	AA				
rs719675	28784203	AA	AB	AB	AB	AB				
rs1522921	28892267	AA	AB	AB	AA	AB				
rs722611	29256331	AA	AA	AA	AA	AA				
rs4087745	29322168	NoCall	NoCall	BB	NoCall	NoCall				
rs1362355	29347428	AB	AA	AA	NoCall	NoCall				
rs1420117	29374581	BB	BB	BB	AB	AB		Average		
	Size (bp)	4044451	3494094	3515023	5632740	3515023	11307603	5 251 489	5.3	cM
								2/g = M	0.053	M
								g =	38.1	generations
									952.1	years

Fig. S1. Array based haplotype analysis in six patients (Pt1-5 and Pt9), showing the size of the homozygous regions (Pt1-5) 4044451 bp, 3494094 bp, 3515023 bp, 5632740 bp, 4628862 bp, and 4628862 bp, respectively. Pt 9 showed homozygosity between marker rs477644 and rs6462058, corresponding to 11307603 bp. The linkage disequilibrium extends over a mean homozygous region of 5.3 cM, which equals 38 meioses/generations.

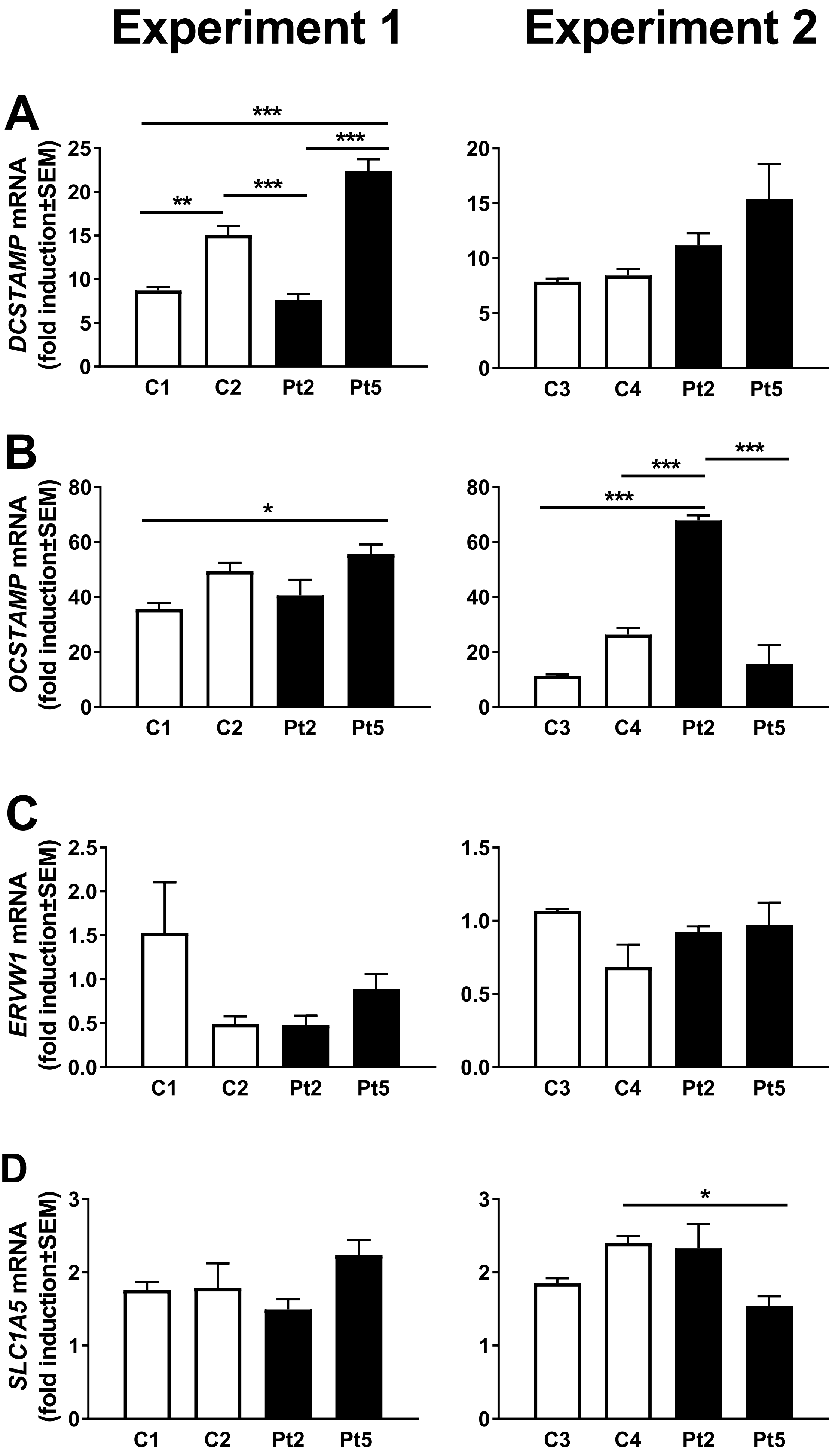

Fig. S2. Gene expression in osteoclast from patients (Pt2, Pt5), and controls (C1, C2, C3, C4). Fold induction of gene expression between cells treated with M-CSF (M) with and without RANKL (RL) for three days. All values are given as mean ±SEM (n=4). * $P \leq 0.05$, ** $P \leq 0.005$, *** $P \leq 0.001$ between indicated groups.
